# Supplementary material for: Trigger factor both holds and folds its client proteins
Source: Nat Commun. 2022 Jul 15;13:4126. doi: 10.1038/s41467-022-31767-6 (PMC9287376; doi:10.1038/s41467-022-31767-6)
Supplement: Supplementary file 1 — Supplementary Information [file 41467_2022_31767_MOESM1_ESM.pdf]

Supplementary Information

Trigger factor both holds and folds its client proteins

Kevin Wu ***et al*****.**

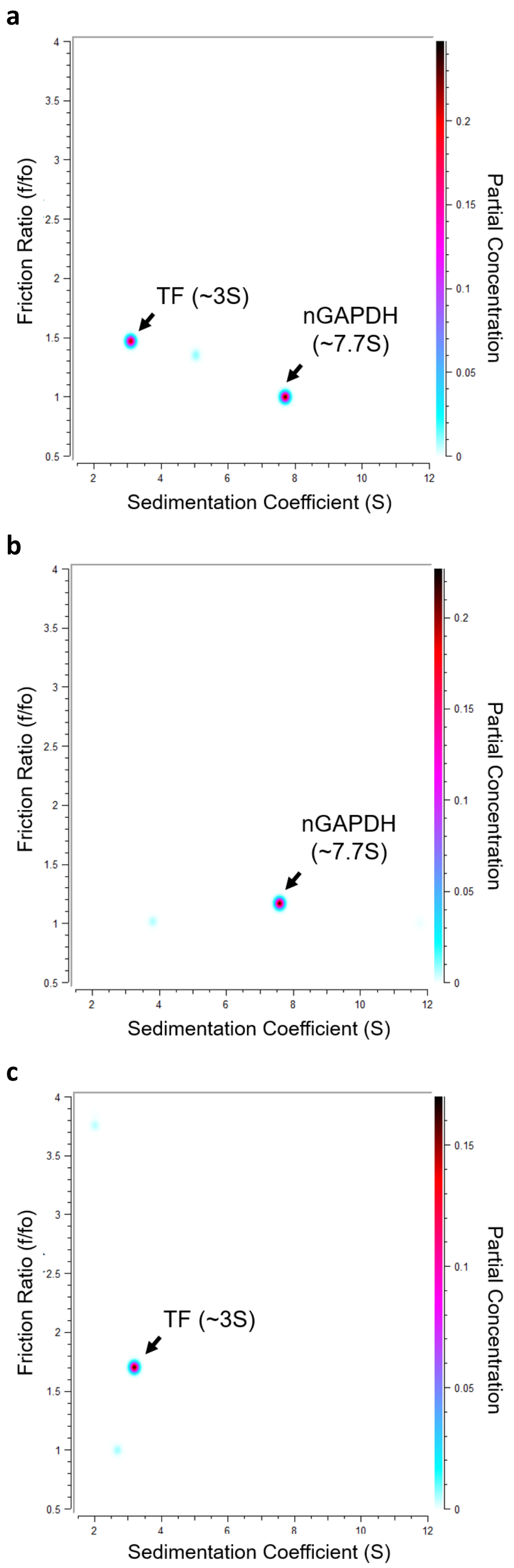

Supplementary Fig. 1 Analytical ultracentrifugation.

Analytical ultracentrifugation analysis of (a) a mixture of native GAPDH and trigger factor, (b) native GAPDH, and (c) trigger factor. All the experiments were conducted in buffer A containing 0.26 M GdnHCl, 4°C.

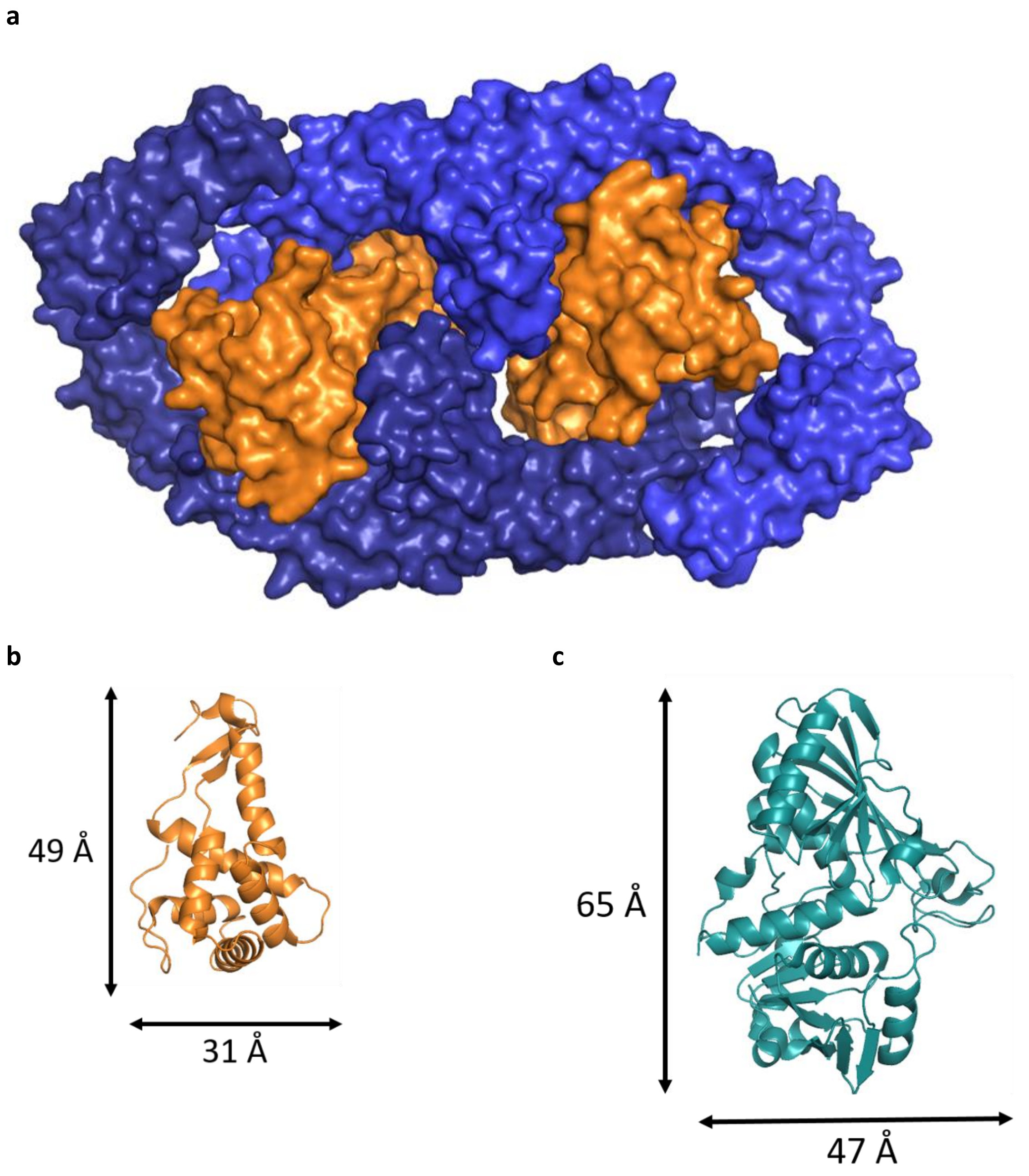

Supplementary Fig. 2 Crystal structure of trigger factor-S7 complex
a Crystal structure of *T. maritima* trigger factor-S7 2:2 complex (pdb:3gty) [1]. Trigger factor molecules are colored in blue while S7 molecules are colored in orange.
b Structure of *T. maritima* S7 (pdb: 3gty).
c Structure of *O. cuniculus* (rabbit) GAPDH (pdb: 1j0x). Only a single monomer is shown.

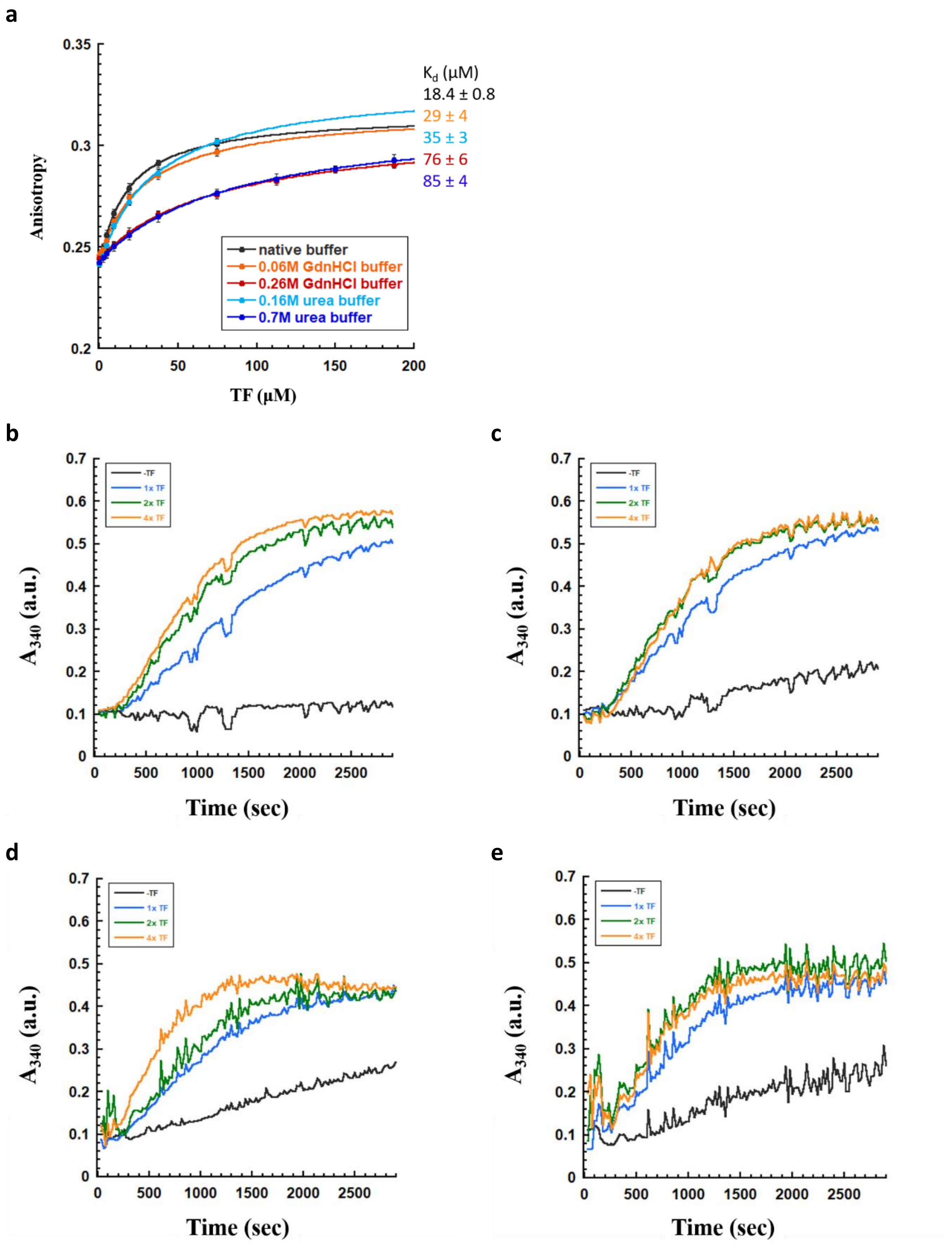

Supplementary Fig. 3 The effects of denaturants on the chaperone activity of trigger factor.

a Fluorescence anisotropy titration curves of fluorescently labelled trigger factor that was titrated with increasing concentrations of non-labelled trigger factor. All experiments were performed in buffer A at 4°C. The data points were fitted to a hyperbola equation to determine the dissociation constant (K_d). Each anisotropy measurement is the average of

twenty independent measurements, and the error reported in the plot is the standard deviation. Values of K_d reported are the mean ± s.e.m. of the fit.

b-c 32 μM GdnHCl-denatured GAPDH was diluted 11.5-fold (b) or 50-fold (c) into buffer A containing various concentrations of trigger factor (0, 2.78, 5.56, 11.12 μM). Samples were incubated on ice for 3 hours before measuring the enzymatic activity of GAPDH. The final concentrations of GdnHCl were 0.26 M (b) and 0.06 M (c). a.u.: arbitrary units.

d-e 32 μM urea-denatured GAPDH was diluted 11.5-fold (d) or 50-fold (e) into the buffer A containing various concentrations of trigger factor (0, 2.78, 5.56, 11.12 μM). Samples were incubated on ice for 3 hours before measuring the enzymatic activity of GAPDH. The final concentration of urea was 0.7 M (d) and 0.16 M (e). a.u.: arbitrary units.

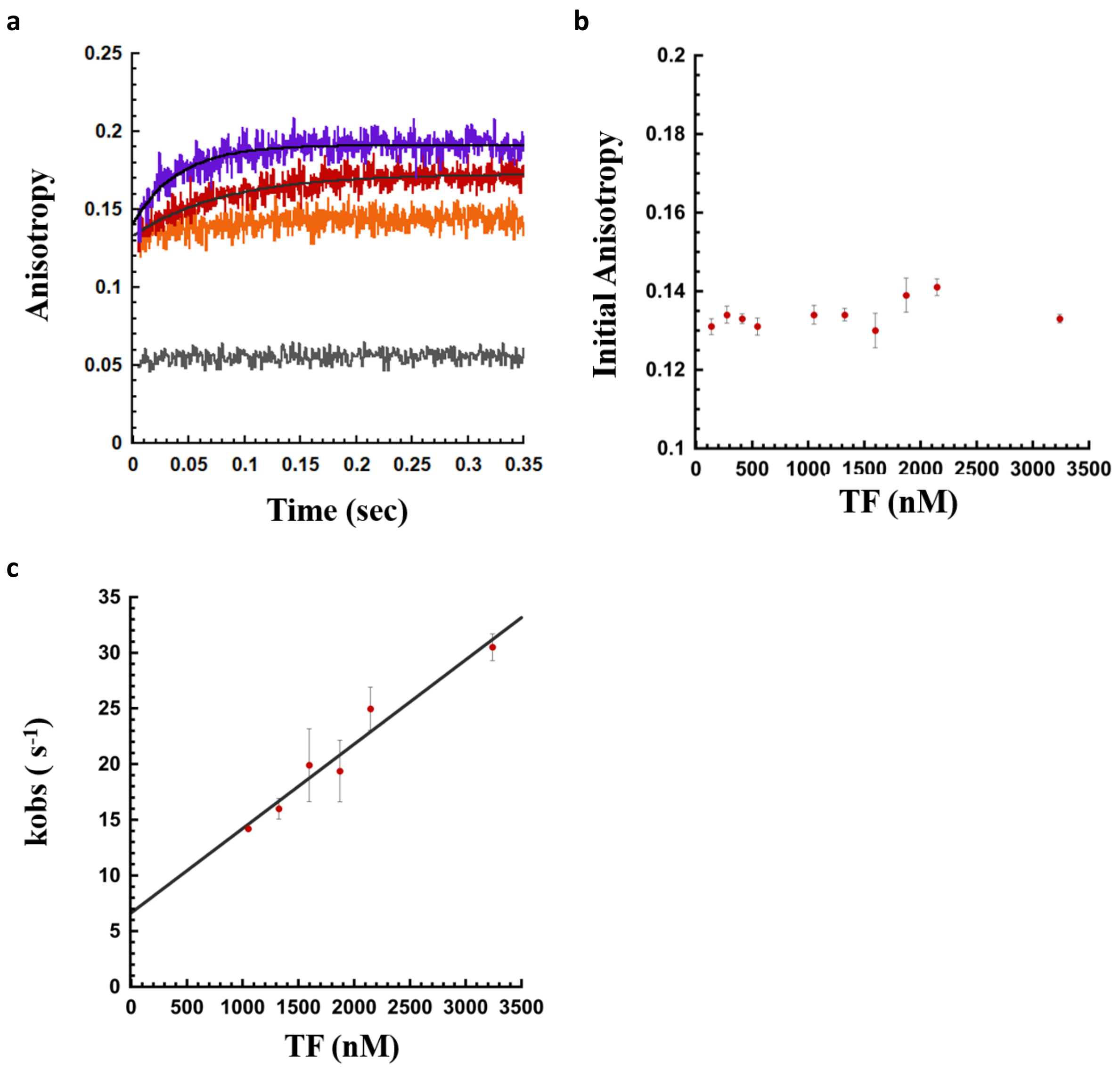

Supplementary Fig. 4 Kinetics of trigger factor binding to refolding GAPDH.

a Changes in fluorescence anisotropy of 0.13 µM refolding GAPDH upon association with trigger factor at 4°C. The concentration of trigger factor after mixing was 0.13-3.24 µM. For the purpose of clarity, only three kinetic traces with 0 µM (orange), 0.4 µM (red) and 2.1 µM (purple) of trigger factor are shown in the graph. The kinetic traces were fitted to a single-exponential function. The trace for denatured GAPDH diluted into the denatured buffer is shown in dark grey line.

b Initial anisotropy values were calculated from the fit of kinetic traces and plotted as a function of trigger factor concentrations. The Initial anisotropy of three independent kinetic traces, averages of ten replicates, were plotted to show the experimental error.

c Change in observed rate constant (k_{obs}) with trigger factor concentration. The k_{obs} increased linearly with the concentration of trigger factor, suggesting a single-step bimolecular reaction. Fitting the plot to a linear curve, an association rate constant (k_{on}) of 7.6 $*10^6$ M^{-1} s^{-1} can be obtained from the slope and dissociation rate constant (k_{off}) of 6.7 s^{-1} can be obtained from the y-intercept. The kobs of three independent kinetic traces, averages of ten replicates, were plotted to show the experimental error.

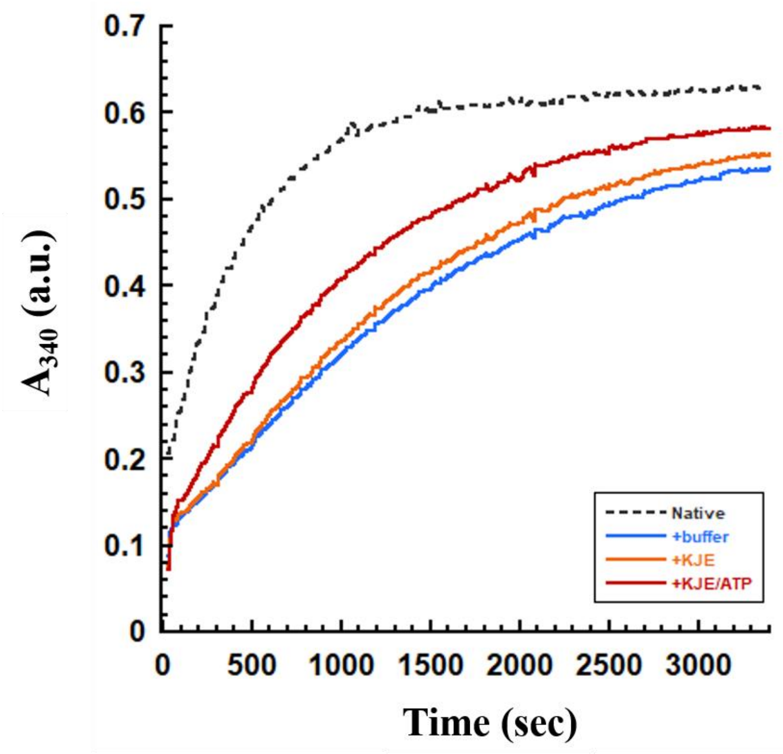

Supplementary Fig. 5 The effect of DnaKJE on trigger factor-induced GAPDH refolding.
2.78 µM refolding GAPDH was incubated with 11.12 µM trigger factor on ice for 3 hours. The refolding mixtures were then 1:1 mixed with buffer A or buffer containing the DnaKJE system with or without ATP. Refolding solutions were continued to incubate at room temperature for 1 hour before measuring the GAPDH enzymatic activity. The relative activity of GAPDH can be further enhanced from ~35% to ~50% by adding DnaKJE. The relative activity was calculated by comparing the yield of active refolding GAPDH with the same amount of native GAPDH (without denaturation; black dash line). The amount of active GAPDH is proportional to the slope of the trace within the linear range (50-350 sec). a.u.: arbitrary units.

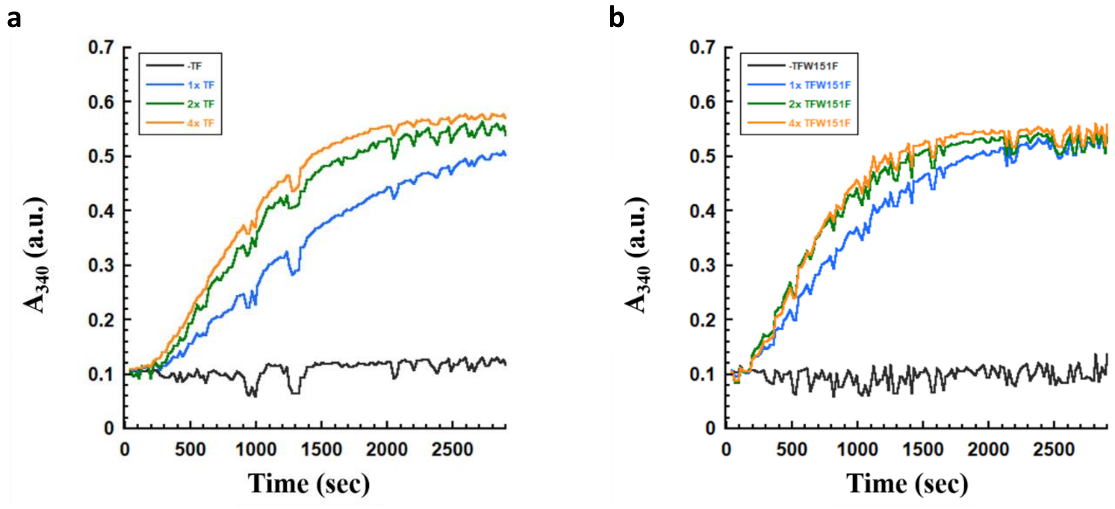

Supplementary Fig. 6 The comparison of GAPDH refolding in the presence of wild-type trigger factor (a) and trigger factor W151F.

32 µM GdnHCl-denatured GAPDH was diluted 11.5-fold into buffer A containing various concentrations of wild-type trigger factor (a) or trigger factor W151F variant (b). Samples were incubated on ice for 3 hours before measuring the GAPDH enzymatic activity. Note that panel a reproduced from Supplementary Fig. 3b to enable easy comparison with TFW151F data. a.u.: arbitrary units.

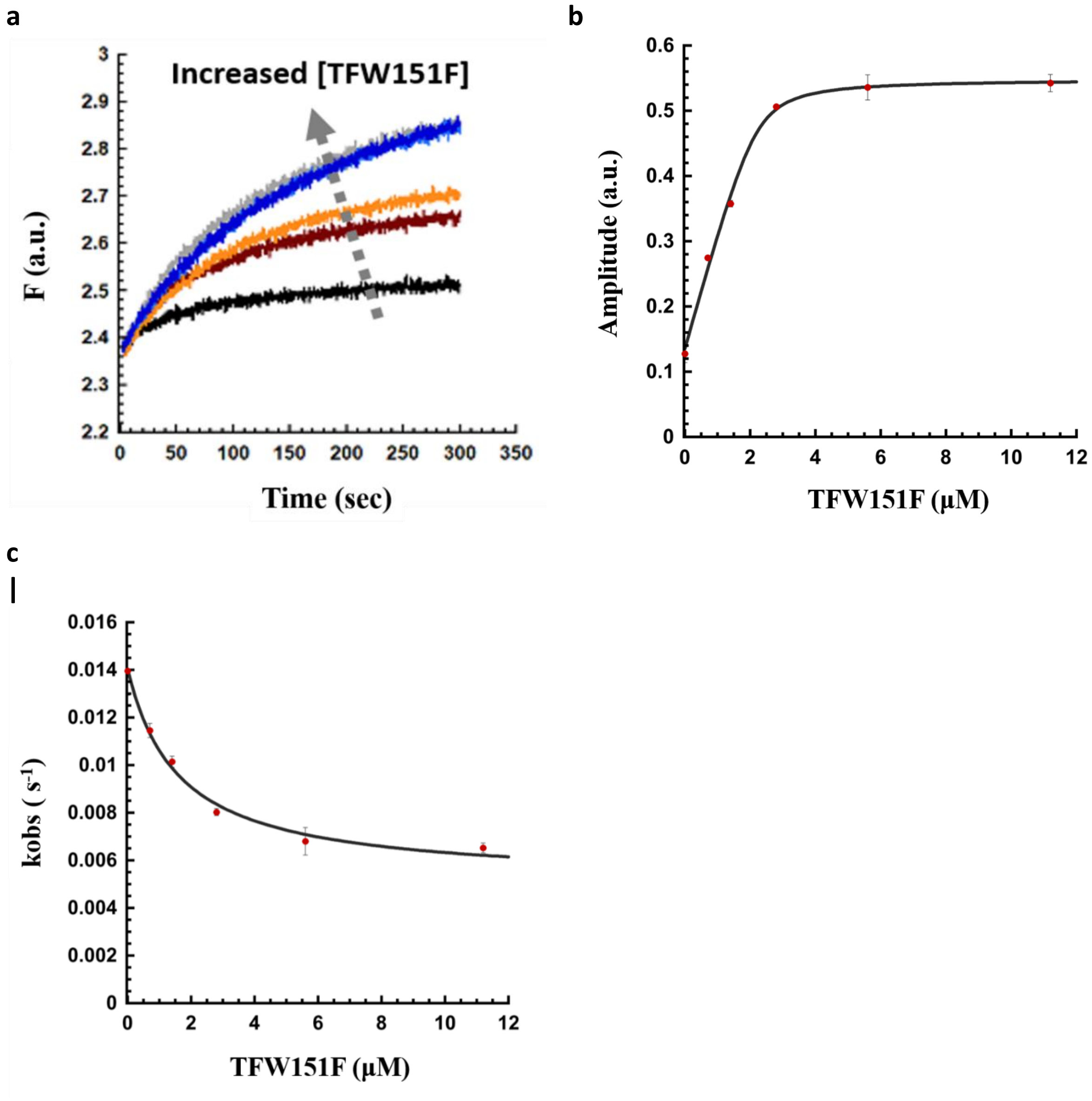

Supplementary Fig. 7 Kinetics of GAPDH folding in the presence of trigger factor W151F variant.

a Changes in intrinsic fluorescence of 1.4 µM refolding GAPDH in the presence of various concentrations of trigger factor W151F (0-11.2 µM after mixing) at 4°C. All traces can be well described by a single-exponential function. a.u.: arbitrary units.

b Plot of the amplitude of the fluorescence trace versus the concentration of trigger factor W151F. The amplitude increases with concentrations of trigger factor W151F, indicating that trigger factor stabilizes an intermediate state of GAPDH. The amplitude of kinetic traces is the average of four to five independent traces, and the error reported in the plot is the standard deviation. The data points were fitted to a quadratic equation. a.u.: arbitrary units.

c Plot of observed rate constant (k_{obs}) for GAPDH refolding as a function of trigger factor concentration. The k_{obs} decreases to a non-zero value at high concentrations of trigger factor, suggesting that GAPDH could refold to this intermediate state while bound to trigger factor. The kobs of kinetic traces is the average of four to five independent traces, and the error reported in the plot is the standard deviation. The data points were fitted to a negative hyperbola equation.

a

b

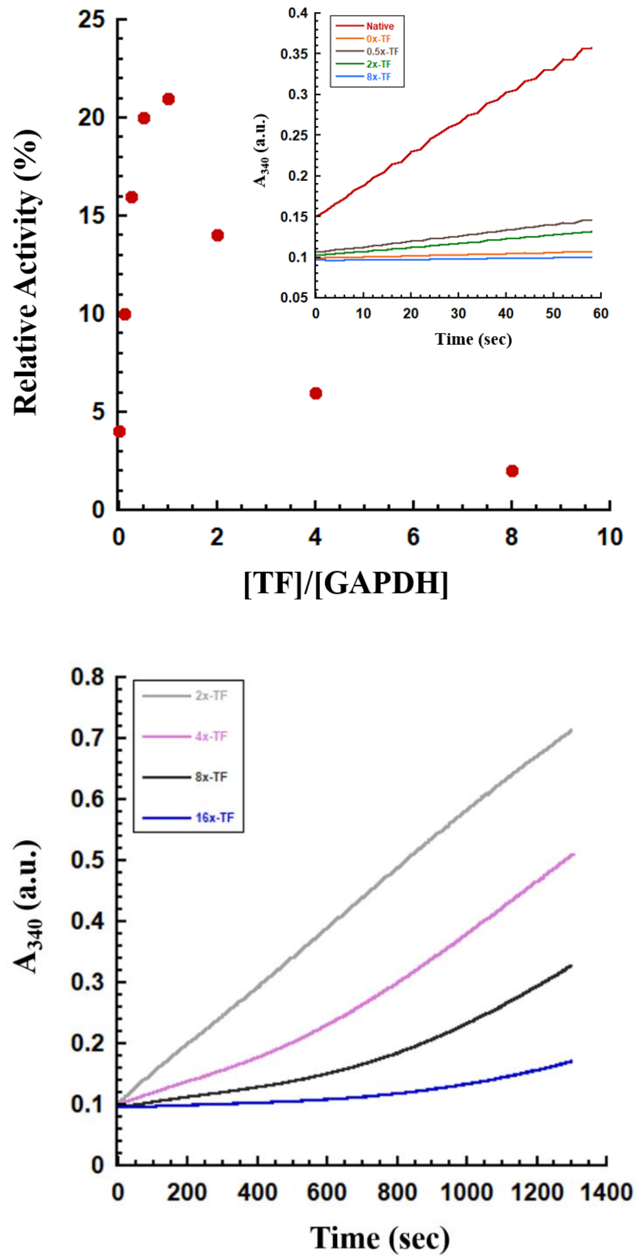

Supplementary Fig. 8 The effects of trigger factor on the refolding of GAPDH at 25°C

(a) 2.78 µM refolding GAPDH was incubated at 25°C in the presence of various concentrations of trigger factor (0-44.5 µM). The reactivation yield was increased as trigger factor concentration increased and reached a maximum value at 1:1 ratio of GAPDH and trigger factor. Further increasing concentrations of trigger factor decreases the refolding yield of GAPDH. Inset: the kinetic trace of GAPDH refolding in various concentrations of trigger factor. These traces were monitored in ~1 minute and the slope of the trace is proportional to the amount of active GAPDH formed in solutions containing 0.06 M GdnHCl. The relative activity was calculated by comparing the slope between refolding GAPDH and the same amount of native GAPDH (without denaturation). a.u.: arbitrary units.

(b) These kinetic traces were measured in the same experiment as described above but each trace was recorded in ~20 minutes. There is a clear lag phase prior to the GAPDH activity fully recovered. a.u.: arbitrary units.

a

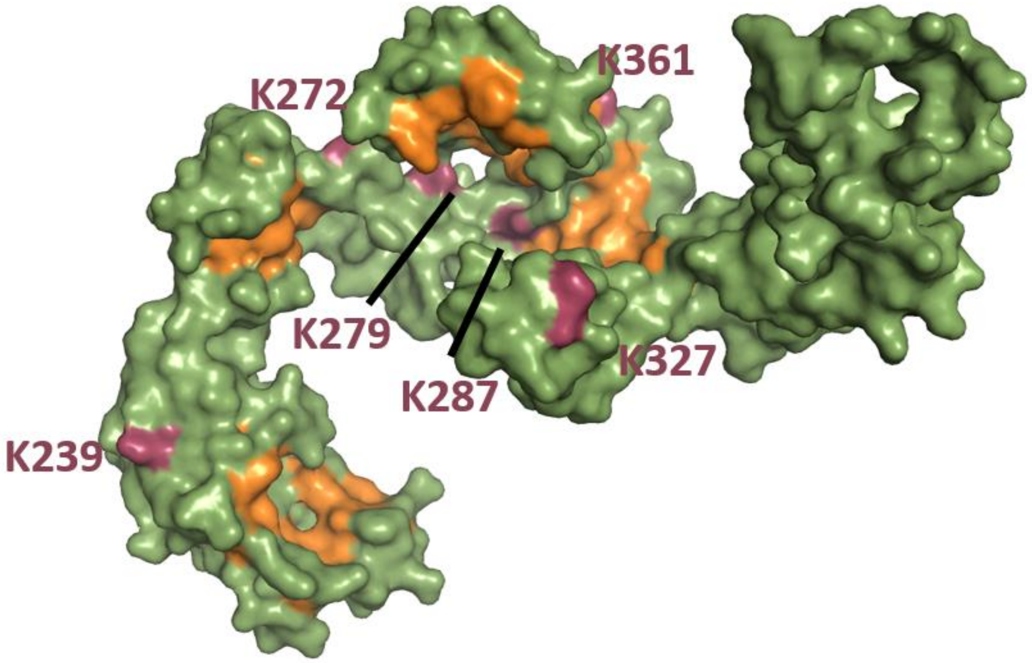

b

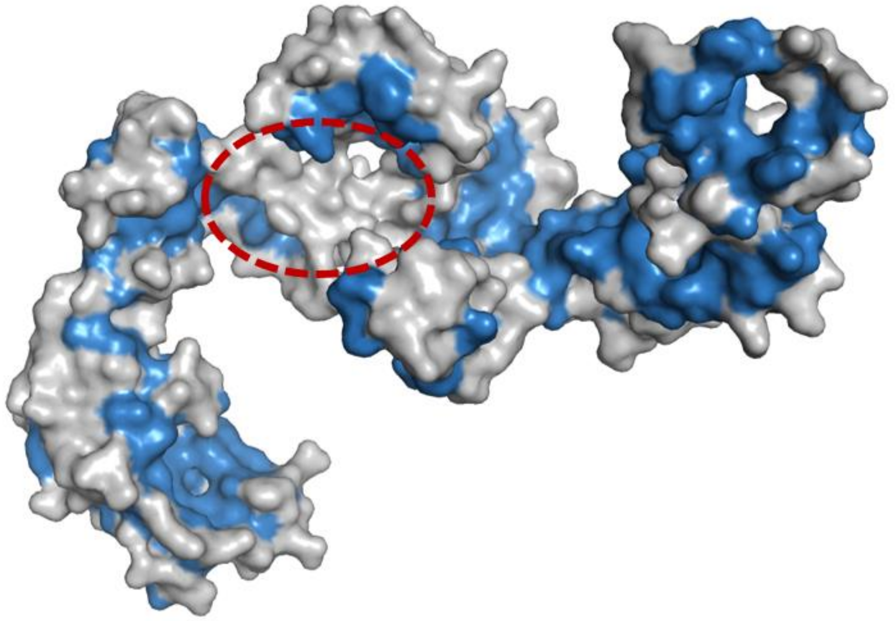

Supplementary Fig. 9 Comparison of XL-MS data with substrate binding sites and hydrophobic residues.

a Comparison of the substrate binding site identified by NMR studies with PhoA as a client [2] and XL-MS data with GAPDH as a client (this paper). The cross-linked sites, colored in raspberry, are mapped onto the crystal structure of trigger factor (pdb: 1w26). The substrate binding sites identified by previous NMR studies are colored in orange. The orientation of structure is identical to the one in Fig. 4c.

b Hydrophobic residues on trigger factor are colored in blue. The hydrophilic region at the bottom of the trigger factor cradle that we identify as interacting with GAPDH (residues 275-290) is indicated with a red dashed circle.

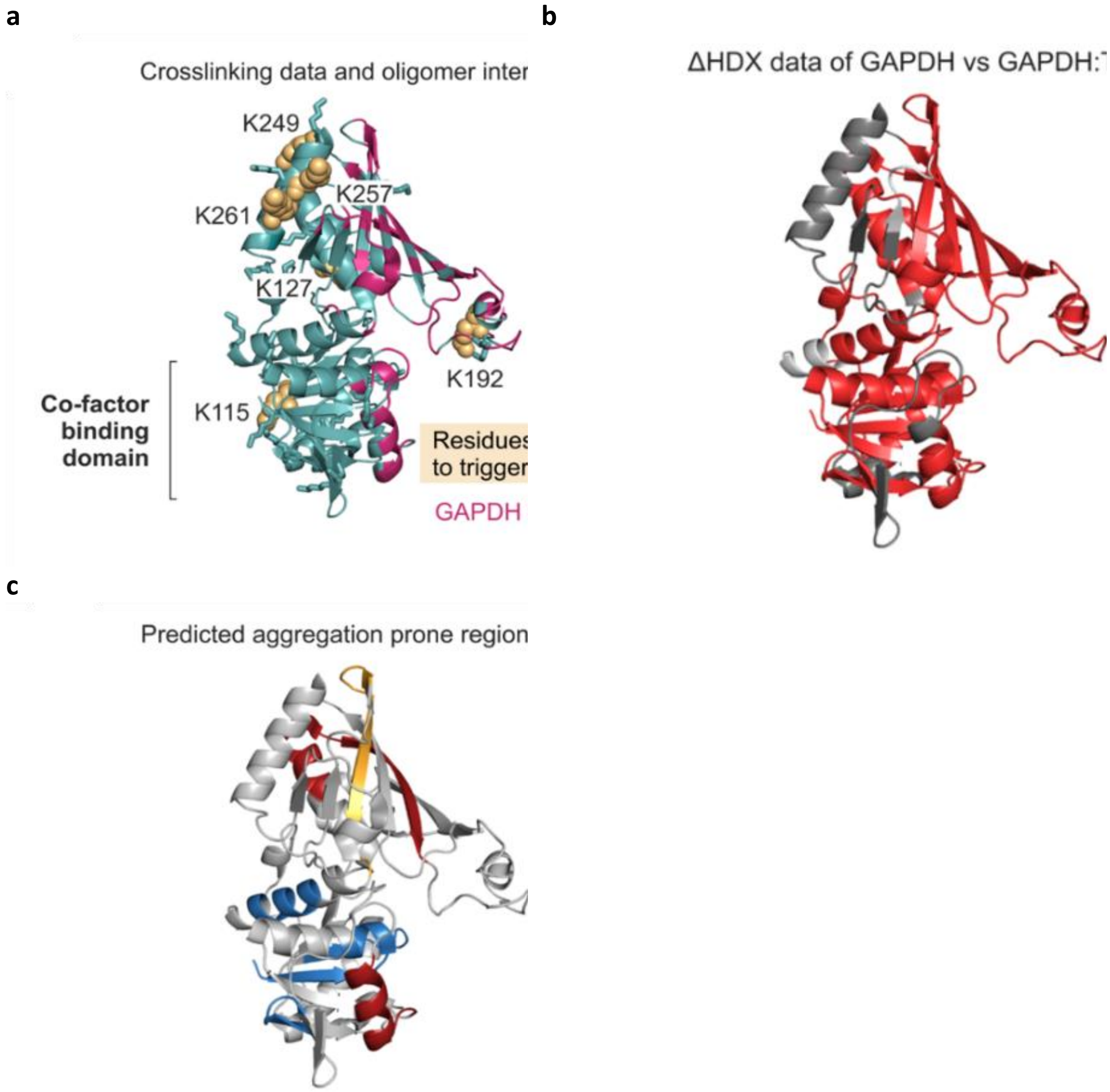

Supplementary Fig. 10 Comparison of XL-MS and HDX-MS data with oligomer interfaces and aggregation prone regions.

a Sites on trigger factor that crosslink to GAPDH, shown as pale-yellow spheres, are labelled and mapped onto a subunit of crystal structure of rabbit-muscle GAPDH (pdb: 1J0X). The oligomeric interfaces of native GAPDH are colored in pink. Two structural domains of GAPDH, catalytic domain and cofactor-binding domain, are labelled. Non-cross-linked lysine residues are shown as sticks.

b The differences in deuterium uptake are plotted on the structure of GAPDH (right) PDB: 1J0X. Red regions are deprotected from exchange when bound to trigger factor.

c Predicted aggregation prone regions on GAPDH (PDB: 1J0X) determined using AGGRESCAN [3]. Note that panels (a) and (b) reproduced from Fig. 4d and 5d in the main text to enable easy comparison with the AGGRESCAN data.

Supplementary Table 1 List of intermolecular and intramolecular crosslinked peptides detected in the trigger factor:GAPDH complex. The identity of the crosslinked peptides for each pair is shown, along with the residue numbering in the protein and the position of the crosslinked site in the peptide.

Peptide 1					Peptide 2				
**Sequence**	**Protein**	**From**	**To**	**Linkage Position**	**Sequence**	**Protein**	**From**	**To**	**Linkage Position**
**Intermolecular crosslinks**									
AVGKVIPELDGK	GAPDH	214	225	K4	TNELKADEER	TF	357	366	K5
KVEER	TF	239	243	K1	LEKAAK	GAPDH	247	252	K3
VVKQASEGPLK	GAPDH	259	269	K3	KVEER	TF	239	243	K1
KNMER	TF	272	276	K1	LEKAAK	GAPDH	247	252	K3
ELKSAIR	TF	277	283	K3	YDDIKK	GAPDH	253	258	K5
VKSQAIEGLVK	TF	286	296	K2	LEKAAK	GAPDH	247	252	K3
FGGNEKQALELPR	TF	322	334	K6	GGAKR	GAPDH	112	116	K4
FGGNEKQALELPR	TF	322	334	K6	YDDIKK	GAPDH	253	258	K5
FGGNEKQALELPR	TF	322	334	K6	GGAKR	GAPDH	112	116	K4
FGGNEKQALELPR	TF	322	334	K6	LEKAAK	GAPDH	247	252	K3
FGGNEKQALELPR	TF	322	334	K6	YDDIKK	GAPDH	253	258	K5
FGGNEKQALELPR	TF	322	334	K6	VVKQASEGPLK	GAPDH	259	269	K3
FGGNEKQALELPR	TF	322	334	K6	TVDGPSGKLWR	GAPDH	185	195	K8
ELFEEQAKR	TF	335	343	K8	LEKAAK	GAPDH	247	252	K3
TNELKADEER	TF	357	366	K5	LEKAAK	GAPDH	247	252	K3
TNELKADEER	TF	357	366	K5	YDDIKK	GAPDH	253	258	K5
**Intramolecular crosslinks**									
VKSQAIEGLVK	TF	286	296	K2	ELKSAIR	TF	277	283	K3
KGKVPMNIVAQR	TF	46	57	K1	SELVNVAKK	TF	30	38	K8
GKVPMNIVAQR	TF	47	57	K2	SELVNVAKK	TF	30	38	K8
EKINPAGAPTYVPGEYK	TF	82	98	K2	TNELKADEER	TF	357	366	K5

EKINPAGAPTYVPGEYK	TF	82	98	K2	TNELKADEER	TF	357	366	K5
EKINPAGAPTYVPGEYK	TF	82	98	K2	NVALEEQAVEAVLAKAK	TF	400	416	K15
QQATWKEK	TF	147	154	K6	KVEER	TF	239	243	K1
FGGNEKQALELPR	TF	322	334	K6	SELVNVAKK	TF	30	38	K8
VKGLIEEMASAYEDPK	TF	367	382	K2	ELKSAIR	TF	277	283	K3
NVALEEQAVEAVLAKAK	TF	400	416	K15	TNELKADEER	TF	357	366	K5
TNELKADEER	TF	357	366	K5	ELKSAIR	TF	277	283	K3
TNELKADEER	TF	357	366	K5	NKELMDNMR	TF	391	399	K2
TNELKADEER	TF	357	366	K5	VKSQAIEGLVK	TF	286	296	K2
TNELKADEER	TF	357	366	K5	NKELMDNMR	TF	391	399	K2
FGGNEKQALELPR	TF	322	334	K6	VKSQAIEGLVK	TF	286	296	K2
FGGNEKQALELPR	TF	322	334	K6	TNELKADEER	TF	357	366	K5
FGGNEKQALELPR	TF	322	334	K6	ELKSAIR	TF	277	283	K3
TVDGPSGKLWR	GAPDH	185	195	K8	LEKAAK	GAPDH	247	252	K3
VVKQASEGPLK	GAPDH	259	269	K3	LEKAAK	GAPDH	247	252	K3

Supplementary Table 2 HDX Data Summary Table. SD = standard deviation, CI = confidence interval. A one-tailed t-test was used.

Data Set	TF	GAPDH	TF GAPDH
**HDX reaction details**	10 mM potassium phosphate, pD 7.6, 0.1M KCl, 1mM TCEP and 0.26M GdnHCl, 4 ºC	10 mM potassium phosphate, pD 7.6, 0.1M KCl, 1mM TCEP and 0.26M GdnHCl, 4 ºC	10 mM potassium phosphate, pD 7.6, 0.1M KCl, 1mM TCEP and 0.26M GdnHCl, 4 ºC
**HDX time course (min)**	0.5, 2, 5, 10 or 30 min		
**HDX control samples**	Maximally labeled controls were not performed.		
**Back-exchange**	~ 30 %		
**# of Peptides**	171	97	TF 172<br>GAPDH 96
**Sequence coverage**	93.75%	79.58%	TF 93.75%<br>GAPDH 79.58%
**Average peptide length / Redundancy**	9.29 / 3.92	8.89 / 3.25	TF 9.28 / 3.94<br>GAPDH 8.89 / 3.23
**Replicates (biological or technical)**	3 (technical)	3 (technical)	3 (technical)
**Repeatability**	0.055 (average SD)	0.052 (average SD)	TF 0.061<br>GAPDH 0.063 (average SD)
**Significant differences in HDX (delta HDX > X D)**	Reference	Hybrid Significance test: 99% CI: 0.31Da/ p-value <-0.01	Hybrid Significance test: 99% CI: 0.31Da/ p-value <-0.01

Supplementary References:

1 Martinez-Hackert, E. & Hendrickson, W. A. Promiscuous substrate recognition in folding and assembly activities of the trigger factor chaperone. *Cell* **138**, 923-934, doi:10.1016/j.cell.2009.07.044 (2009).

2 Saio, T., Guan, X., Rossi, P., Economou, A. & Kalodimos, C. G. Structural basis for protein antiaggregation activity of the trigger factor chaperone. *Science* **344**, 1250494, doi:10.1126/science.1250494 (2014).

3 Conchillo-Sole, O. *et al.* AGGRESCAN: a server for the prediction and evaluation of "hot spots" of aggregation in polypeptides. *BMC Bioinformatics* **8**, 65, doi:10.1186/1471-2105-8-65 (2007).
