# Supplementary material for: Trigger factor both holds and folds its client proteins
Source: Nat Commun. 2022 Jul 15;13:4126. doi: 10.1038/s41467-022-31767-6 (PMC9287376; doi:10.1038/s41467-022-31767-6)
Supplement: Supplementary file 3 — Description of additional Supplementary File [file 41467_2022_31767_MOESM3_ESM.pdf]

Descriptions of additional Supplementary data files

Supplementary Data 1 : Reporting summary for XL-MS experiment design and analysis based on community guidelines
