# Supplementary material for: Trigger factor both holds and folds its client proteins
Source: Nat Commun. 2022 Jul 15;13:4126. doi: 10.1038/s41467-022-31767-6 (PMC9287376; doi:10.1038/s41467-022-31767-6)
Supplement: Supplementary file 5 — Reporting Summary [file 41467_2022_31767_MOESM5_ESM.pdf]

nature portfolio

Corresponding author(s): James Bardwell; Antonio N. Calabrese

Last updated by author(s): Jun 7, 2022

Reporting Summary

Nature Portfolio wishes to improve the reproducibility of the work that we publish. This form provides structure for consistency and transparency in reporting. For further information on Nature Portfolio policies, see our Editorial Policies and the Editorial Policy Checklist.

Statistics

For all statistical analyses, confirm that the following items are present in the figure legend, table legend, main text, or Methods section.

n/a	Confirmed	
☐	☒	The exact sample size (*n*) for each experimental group/condition, given as a discrete number and unit of measurement
☐	☒	A statement on whether measurements were taken from distinct samples or whether the same sample was measured repeatedly
☐	☒	The statistical test(s) used AND whether they are one- or two-sided<br>*Only common tests should be described solely by name; describe more complex techniques in the Methods section.*
☒	☐	A description of all covariates tested
☒	☐	A description of any assumptions or corrections, such as tests of normality and adjustment for multiple comparisons
☐	☒	A full description of the statistical parameters including central tendency (e.g. means) or other basic estimates (e.g. regression coefficient) AND variation (e.g. standard deviation) or associated estimates of uncertainty (e.g. confidence intervals)
☐	☒	For null hypothesis testing, the test statistic (e.g. *F*, *t*, *r*) with confidence intervals, effect sizes, degrees of freedom and *P* value noted<br>*Give P values as exact values whenever suitable.*
☒	☐	For Bayesian analysis, information on the choice of priors and Markov chain Monte Carlo settings
☒	☐	For hierarchical and complex designs, identification of the appropriate level for tests and full reporting of outcomes
☒	☐	Estimates of effect sizes (e.g. Cohen's *d*, Pearson's *r*), indicating how they were calculated

Our web collection on statistics for biologists contains articles on many of the points above.

Software and code

Policy information about availability of computer code

Data collection	HDX-MS and XL-MS data were acquired using software supplied by the instrument vendors, MassLynx software (Waters) for HDX-MS data and Xcalibur (Thermo) for XL-MS data.
Data analysis	KaleidaGraph (v4.5); UltraScan (v4.0); PLGS (v3.0.2); DynamX (v3.0.0); Deuteros (v2.0); MeroX (v2.0.1.4);

For manuscripts utilizing custom algorithms or software that are central to the research but not yet described in published literature, software must be made available to editors and reviewers. We strongly encourage code deposition in a community repository (e.g. GitHub). See the Nature Portfolio guidelines for submitting code & software for further information.

Data

Policy information about availability of data

All manuscripts must include a data availability statement. This statement should provide the following information, where applicable:

- Accession codes, unique identifiers, or web links for publicly available datasets
- A description of any restrictions on data availability
- For clinical datasets or third party data, please ensure that the statement adheres to our policy

MS data have been deposited to the ProteomeXchange Consortium via the PRIDE partner repository with the dataset identifiers PXD029365 (XL-MS) and PXD029364 (HDX-MS). All other data are available from the corresponding author upon reasonable request.

Field-specific reporting

Please select the one below that is the best fit for your research. If you are not sure, read the appropriate sections before making your selection.

- [x] Life sciences
- [ ] Behavioural & social sciences
- [ ] Ecological, evolutionary & environmental sciences

For a reference copy of the document with all sections, see nature.com/documents/nr-reporting-summary-flat.pdf

Life sciences study design

All studies must disclose on these points even when the disclosure is negative.

Sample size	Sample sizes were chosen based on convention in the field. These sample sizes were sufficient given the robust signal changes measured in the experiments.
Data exclusions	No data were excluded.
Replication	The experimental findings were reliably reproduced. All the experiments have been repeated at least three times.
Randomization	There were no experiments that necessitated randomization, since the original samples were only one type of protein at a time in a buffer. The samples were aliquots drawn from a larger batch of purified proteins. No further randomization was possible.
Blinding	Blinding is necessary when manipulation of analysis steps can lead to different conclusions from the analysis. All analysis done herein was curve-fitting by a computer algorithm with no human input, therefore, no blinding was necessary.

Reporting for specific materials, systems and methods

We require information from authors about some types of materials, experimental systems and methods used in many studies. Here, indicate whether each material, system or method listed is relevant to your study. If you are not sure if a list item applies to your research, read the appropriate section before selecting a response.

Materials & experimental systems

n/a	Involved in the study	
☒	☐	Antibodies
☒	☐	Eukaryotic cell lines
☒	☐	Palaeontology and archaeology
☒	☐	Animals and other organisms
☒	☐	Human research participants
☒	☐	Clinical data
☒	☐	Dual use research of concern

Methods

n/a	Involved in the study	
☒	☐	ChIP-seq
☒	☐	Flow cytometry
☒	☐	MRI-based neuroimaging
